# Very early symptomatic metastasis pseudoprogression after stereotactic brain radiosurgery in a melanoma patient treated with BRAF/MEK inhibitors: a case report and review of the literature

**DOI:** 10.3389/fonc.2024.1449228

**Published:** 2024-10-22

**Authors:** Edouard Romano, Sebastien Tran, Assma Ben Aissa, Miguel Carvalho Goncalves, André Durham, Pelagia Tsoutsou

**Affiliations:** ^1^ Department of Radiation Oncology, University Hospitals of Geneva, Geneva, Switzerland; ^2^ Department of Radiation Oncology, Vaud University Hospital Center, Lausanne, Switzerland; ^3^ Department of Medical Oncology, University Hospitals of Geneva, Geneva, Switzerland

**Keywords:** case report, brain metastasis, BRAF/MEK inhibitor, stereotactic radiotherapy, pseudoprogression

## Abstract

**Introduction:**

Significant therapeutic changes have recently occurred in the management of melanoma brain metastases (BMs), both in the field of local treatments, with the rise of stereotactic radiotherapy (RT), as well as in systemic ones, with the advent of immunotherapy and targeted therapies (TT). These advances have brought about new challenges, particularly regarding the potential interactions between new TT (notably BRAF/MEK inhibitors) and irradiation. Through a clinical case, we will discuss a side effect not previously described in the literature: ultra-early pseudoprogression (PP) following brain stereotactic radiosurgery (SRS), in a patient treated with dabrafenib-trametinib.

**Case presentation:**

A 61-year-old patient with BRAFV600E-mutated melanoma, receiving second-line dabrafenib-trametinib therapy, was referred for SRS on three progressing meningeal implants, without evidence of systemic progression. Four days after the first RT session (1x6 Gy on a fronto-orbital lesion prescribed 5x6 Gy, and 1x20 Gy single fraction on the other lesions), the patient presented with an epileptic seizure. An MRI, compared to the planning MRI ten days earlier, revealed significant progression of the irradiated lesions. The patient’s condition improved with dexamethasone and levetiracetam, and RT was halted out of caution. A follow-up MRI at one month demonstrated a size reduction of all treated lesions. Subsequent imaging at five months revealed further shrinking of the two lesions treated with an ablative dose of 20 Gy, while the under-treated fronto-orbital lesion progressed. These dynamics suggest an initial PP in the three irradiated lesions, followed by good response in the ablatively treated lesions and progression in the partially treated lesion.

**Conclusion:**

To our knowledge, this represents the first documented case of ultra-early PP following brain SRS in a patient receiving concomitant dabrafenib-trametinib. It highlights the need for particular vigilance when using tyrosine kinase inhibitors (TKIs) with SRS, and warrants further research into potential treatment interactions between RT and novel systemic agents, as well as the optimal treatment sequence of melanoma BMs.

## Introduction

Brain metastases (BMs) are an increasingly complex and important topic in cancer management. BMs now occur in 20% of patients with systemic cancers (1), particularly in melanoma, where BM incidence ranges from 10% to 73% (1), with a particular affinity for BRAF-V600 mutated disease (2).

BMs negatively impact survival and quality of life (3). Their management is debated, in particular the role and sequence of local and systemic treatment, as in both modalities, important advances represent attractive options. Central nervous system (CNS) active systemic treatments such as immunotherapy and tyrosine kinase inhibitors have proved a game-changer. Improvements are particularly notable in melanoma, especially in the setting of BRAF-V600E and BRAF-V600K mutations (4), which represent 40-50% of metastatic melanoma cases and are amenable to targeted therapies (TT) with BRAF/MEK inhibitors (BRAF/MEKi) (5, 6).

Progress in radiotherapy (RT) techniques has led to the emergence of brain stereotactic radiosurgery (SRS) as a preferred local treatment option alongside surgery for patients with limited BMs. Thus, whole-brain irradiation, the historical standard, has largely been supplanted by SRS, which offers reduced toxicities and excellent local control (7, 8). The 2021 guidelines from ASCO-SNO-ASTRO (9) (non-melanoma-specific) mention that “SRS, WBRT, and the combination of SRS plus WBRT are all reasonable options for patients with more than four unresected or more than two resected brain metastases and better performance status”, also stating that “SRS may be preferred for patients with better prognosis or where systemic therapy that is known to be active in the CNS is available”. In practice, there is a trend towards an increased number of BMs being treated with SRS (10, 11).

Consequently, SRS is increasingly used in patients also receiving CNS-active systemic treatments. There are however currently few reports regarding the safety and efficacy of approaches combining SRS and novel systemic agents, especially BRAF/MEKi. We will explore a case of very early and symptomatic pseudo-progression (PP) after SRS, not previously described in the literature, and question the possible role of concomitant BRAF/MEKi treatment.

## Case description

A 47-year-old woman presented with a right latero-thoracic superficially spreading melanoma, completely resected in 2008. Histology revealed a melanoma with a Breslow index of 2.4 mm, Clark III, non-ulcerated. In 2011, a subcutaneous relapse of the right breast, considered a metastasis in transit, was excised and identified as a BRAF-V600E mutated melanoma.

In March 2020, she developed multiple site distant metastases involving right axillary and mediastino-hilar lymph nodes, both lungs, and a single intra-axial inferior parietal BM measuring 10 x 9 mm. Systemic treatment with ipilimumab and nivolumab was initiated, without any local treatment for the BM.

In June 2020, after four cycles of dual-immunotherapy, the patient experienced nausea and vomiting. An MRI revealed progression of the contrast-enhancing parietal lesion, from 10 x 9 mm to 18 x 17 mm, with susceptibility-weighted imaging (SWI) hypo-intensity suggestive of bleeding and overall progression of peri-lesional edema. The patient underwent total resection of the lesion. Histopathological analysis showed a densely cellular melanocytic proliferation compatible with her primary melanoma. A chest-abdomen-pelvis CT-scan showed stable extracranial disease.

Postoperative fractionated stereotactic radiosurgery to the surgical cavity was administered concomitantly with the ongoing dual immunotherapy in July 2020, delivering 30 Gy in five fractions of 6 Gy using a HyperArc technique. The irradiation occurred while the patient was on 2 mg daily dexamethasone, prescribed previously and in the process of being tapered. No complications or side effects were reported.

**Figure 1:**
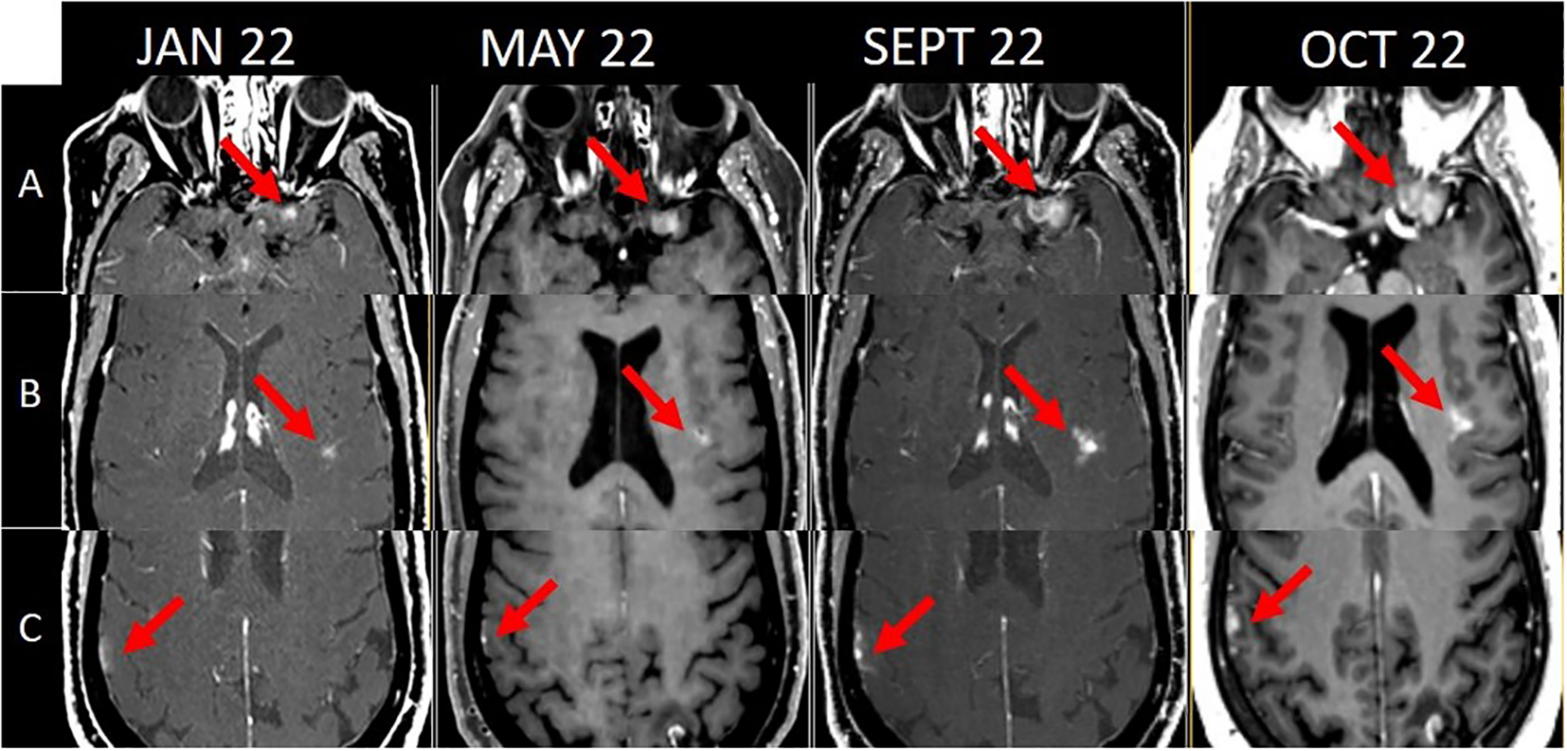
Chronological evolution of the 3 meningeal implants [**(A)** left fronto-orbital, **(B)** left posterior insula, **(C)** right inferior parietal] from diagnosis (January 2022) to SRS (October 2022).

In October 2020, due to pulmonary progression, the systemic treatment was switched to BRAF/MEKi (dabrafenib-trametinib), inducing an almost complete and lasting response (normalization of adenopathy size and near disappearance of pulmonary nodules). Disease control (both systemic and brain) was achieved until January 2022, when three localized meningeal implants (left fronto-orbital, left posterior insula, and right inferior parietal) appeared. At this time, although they were later retrospectively visible, no intracranial new lesion was described. In May, the meningeal implants were first described, with a discrete progression. Without related symptoms or systemic progression, monitoring continued. By September, the nodules progressed further, still without symptoms, other signs of leptomeningeal involvement or systemic progression (**Figure 1**).

The local neuro-oncology tumor board recommended SRS for the three implants. The fronto-orbital lesion measured 18x19x14 mm, the posterior insula 13x8x8 mm and the inferior parietal 13x10x13 mm. Due to its size and proximity to the optic pathways, the fronto-orbital lesion was planned to be treated with five fractions of 6 Gy, while the other two lesions were prescribed with a single fraction of 20 Gy each, all using the CyberKnife platform, with a 70% prescription isodose. The patient was instructed to discontinue BRAF/MEKi one day before treatment due to cautionary evidence on their combined use with SRS. It was however later revealed by herself that this instruction was not followed.

**Figure 2:**
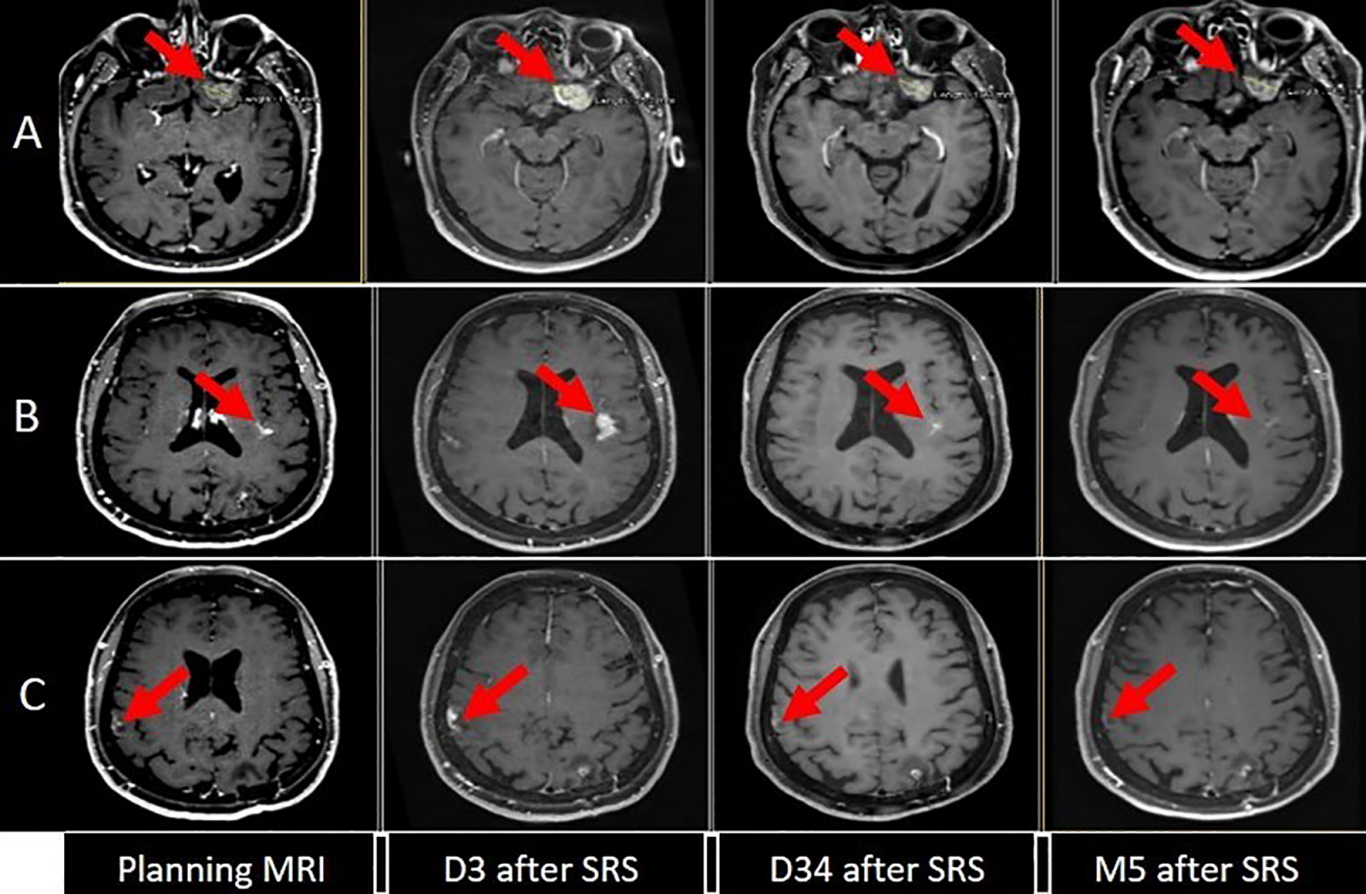
Evolution of brain disease [**(A)** left fronto-orbital, **(B)** left posterior insula, **(C)** right inferior parietal] between planning MRI (one week before SRS), and day 3 (D3), day 34 (D34) and 5 months (M5) after the SRS.

All three lesions were treated as planned on the first RT session. Seventy hours later, the patient experienced mutism and a generalized seizure. An MRI revealed increased size of contrast enhancement, and T2/Flair peripheral edema, with signs of hemorrhage on the right parietal and left insular lesions on the SWI sequence (**Figure 2**).

Under 12 mg of dexamethasone and anti-epileptic treatment, the patient’s neurological condition rapidly improved. Given this unusual situation, the tumor board decided to halt irradiation, thereby not administering the remaining four fractions to the fronto-orbital implant.

An MRI conducted one month after this acute episode revealed regression of all three lesions and almost complete resorption of peritumoral edema, including the fronto-orbital lesion that had only received one fraction of 6 Gy (**Figure 2**).

In March 2023 (five months post-RT), an MRI showed further reduction in size of the two implants treated with ablative SRS (1 x 20 Gy), but progression of the fronto-orbital lesion treated with only 1 x 6 Gy, along with the appearance of a new lesion in the right occipital region (**Figure 2**). Extracranial disease remained stable.

The tumor board recommended SRS on the fronto-orbital and occipital lesions. All lesions were treated in a single fraction using the CyberKnife with a 70% prescription isodose. A dose of 20 Gy was delivered to the new occipital lesion, while 18 Gy were administered to the previously partially treated fronto-orbital lesion. This single fraction regimen was selected to avoid another potential treatment interruption, with a strict dose constraint on the optic path leading to a slight underdosage of the part of the implant closest to that critical structure: the left optic nerve maximum point dose, was 9.6 Gy with a 2 Gy equivalent biological dose of 33.8 Gy on the sum of all plans and 57.8 Gy if a 1 mm margin was applied to the optic nerve. The PTV (GTV + 1 mm) coverage was 87.8% on the 18 Gy isodose and the GTV coverage was 93.2%.

Due to the exceptional event described earlier, dexamethasone (8 mg daily) was prescribed from the day before until two days after SRS. Systemic treatment with dabrafenib-trametinib was continued during SRS, as it was still thought at the time that the previous SRS had taken place with a treatment break. Thus, the hypothesis for the adverse event previously observed was that it was either a PP due to radiation alone, or actual rapid tumor progression due to the suspension of TKIs. Therefore, for the third course, it was decided to continue BRAF/MEKi to avoid the risk of progression, and to give the patient steroids to prevent adverse treatment-induced reactions. No side effects or complications were reported following this irradiation.

In June 2023, the patient developed craniospinal leptomeningeal carcinomatosis (appearance of new supra-tentorial lesions, enhancement within the internal auditory canal bilaterally and diffuse spinal leptomeningeal dissemination, with invasion of the medullary cord in the inferior dorsal region, as well as of the medullary cone), for which a new systemic line of encorafenib and binimetinib was initiated. After an initial response, the patient’s condition deteriorated, and she succumbed to meningeal carcinomatosis within two months. Anti-epileptic treatment was maintained until August 2023, shortly before the patient’s death.

**Table 1** summarizes the timeline and the various interventions performed on the CNS.

**Table 1:** Timeline of SNC procedures.

Timeline	June-July 2020	October 2022	October 2022 D4 after SRS	November 2022 M1 after SRS	March 2023 M5 after SRS	June 2023 M8 after SRS
Clinical status	At time of radiological progression: Nausea/vomiting Postoperatively: fatigue grade 2 and slowed gait, with gradual improvement. ECOG 1 Post-fSRS: no adverse reaction	Neurologically asymptomatic. ECOG 1	Fatigue grade 3, aphasia, generalized seizure	Resolution of symptoms. ECOG 1	At time of radiological findings: no new symptom. ECOG 1 At time of SRS: no new symptom	Altered state of consciousness, confusion
Central nervous system (CNS) imaging	Single BM	-Three new meningeal implants -Known cavity stable	Progression of the three treated lesions	-Shrinkage of the three treated lesions -No new lesion	-Progression of the fronto-orbital lesion -Shrinkage of the two other lesions -One new lesion	Craniospinal lepto-meningeal carcinomatosis
CNS local treatment	Surgery followed by fSRS 5x6 Gy	SRS: 1x20 Gy on two lesions, 5x6 Gy planned for fronto-orbital lesion	Stop RT fronto- orbital lesion after 1 fraction of 6 Gy	No local treatment	SRS: 1x20 Gy on the new lesion, 1x18 Gy on the fronto-orbital lesion	No local treatment
Systemic treatment	-Ipilimumab + Nivolumab -2 mg dexamethasone concurrent to RT	-BRAF/MEKi concurrent to RT -No steroid	-Continuation of BRAF/MEKi -12 mg dexamethasone -Antiepileptic drugs	-BRAF/MEKi -Continuation of anti-epileptic drugs	-BRAF/MEKi -8 mg dexamethasone concurrent to RT -Anti-epileptic drugs	-New systemic line: encorafenib + binimetinib -Anti-epileptic drugs

## Discussion

Pseudo-progression following cerebral SRS is a well-known phenomenon, occurring in approximately 30% of cases, but it’s typically observed from a few weeks to several months post RT (12). PP is believed to result from an immune response triggered by radiation-induced cell death, directed against remaining tumor cells (13). To our knowledge, no case of very early PP, such as the one described here, has been described in the literature. This event is possibly underdiagnosed, as very early follow-up imaging is only performed when worrying symptomatology arises.

Early kinetics of lesions treated with SRS have been poorly explored or understood, as MRIs are usually performed months post-RT. In a study by Peters et al. (12), imaging at six weeks post-treatment showed an increase in size in up to 30% of lesions that subsequently experienced PP with a volume increase over 120% of the pre-treatment volume. Additionally, while 9% of patients exhibited PP across all their lesions, 46% showed a mixture of PP and response, and 46% experienced no PP at all.

In most cases, PP is asymptomatic and is observed on follow-up MRI. In some people however, as in our case report, this PP can lead to various neurological symptoms depending on the location, such as worsening of pre-existing symptoms, symptoms of intracranial hypertension, transient cognitive decline, subacute rhombencephalitis, seizures, somnolence syndrome, endocrine dysfunction or focal deficits (14).

The decision to treat should not be rushed. PP can be defined as an initial contrast enhancement progression followed by regression without any change in oncological treatment. It’s important to first clarify the diagnosis, through multiparametric MRI and/or PET which may indicate PP rather than true progression [no restricted diffusion or higher apparent diffusion coefficient values, low fractional anisotropy, cerebral blood volume reduced < 2 mL/100 g or lesion quotient < 0.3, metabolic activity decreased (15–18)], or by close follow-up, particularly in the absence of symptoms. In our case, a PET was not performed, as it would not have altered our management. The diagnosis of PP was confirmed by the size reduction of lesions on the MRI one month after SRS.

Once PP is diagnosed, treatment is available for symptomatic patients. Generally, drug therapy is the first line, usually initially with corticosteroids. If this treatment fails or is contra-indicated, anti-VEGF agents may be proposed, especially bevacizumab, for which efficacy in treating or preventing PP has been demonstrated in several studies (19–22). In cases of persistent diagnostic doubt or failure of drug treatments, surgery may be proposed. The efficacy of other therapeutic options remains under-documented, so those should be used in cases where standard treatments have failed or are not feasible. These include hyperbaric oxygen therapy (23, 24), laser interstitial thermal therapy (25), anticoagulation (26, 27), combination of pentoxifylline and oral Vitamin E therapy (28), or TKI of VEGF called cediranib (29).

Retrospectively, this PP could hardly have been anticipated. The three meningeal nodules were treated according to the latest ASCO recommendations and the RTOG 90-05 study, based on their size on planning MRI (30, 31). Apart from the BRAF/MEKi and SRS combination, the dose per fraction and the size of the lesions, which is recognized as one of the main risk factor for PP (31–34), no other predictive factor could be identified in our patient. These risk factors include re-irradiation of a lesion (35), V10 of the brain > 10.5 cm^3^ and V12 > 7.9 cm^3^ (36, 37), location on the frontal cortex (38) or deep lesion (39), histology at risk (renal carcinoma, lung adenocarcinoma ALK re-arranged, HER2-amplied breast cancer, and BRAF V600 wild-type melanoma) (35, 40). This data reinforces our warning regarding the association of BRAF/MEKi with SRS, especially as all three lesions, despite their different sizes and fractionation, experienced this PP.

The very rapid onset of PP on all lesions observed in this case following high-dose RT (⩾ 6 Gy) raises questions about a potential interaction between SRS and BRAF/MEKi, although the exact mechanism remains unclear. Evidence remains limited, but previous studies have reported significant combined toxicity of SRS and BRAF inhibitors: in their review, Kroeze et al. (41) reported up to 27% severe combined toxicity (20 out of 75 patients across ten studies), with side effects including intratumoral hemorrhage (11 out of 20 cases) and cerebral edema (7 out of 20 cases) but no early PP. They concluded that caution is necessary when treating with SRS and BRAF/MEKi concomitantly, recommending that TKIs be suspended for SRS delivery whenever possible. Similarly, the ECOG consensus guidelines recommends stopping BRAF/MEKi three days before and after fractionated radiotherapy and one day before and after SRS (42).

In our case, it’s noteworthy that during the second SRS in 2023, no symptomatic PP was reported, despite the concomitant dabrafenib-trametinib treatment. The administration of high-dose dexamethasone may have played a protective role and contributed to this outcome, as steroids are known to effectively manage radiation side effects (43). From our experience, corticosteroids could effectively prevent symptomatic PP in patients where TT must be administered concurrently with SRS, especially when experience with the combination is limited or cautionary. Another potential prophylactic intervention would be the addition of anti-epileptics. Wali’s review (44) suggests that SRS is not associated with excess risk of epilepsy. They suggest however, that in at-risk patients, this prophylaxis could be discussed. Thus, the additional risk of association between TKIs and SRS could lead to discussion of prophylactic epileptic drugs in selected cases.

Driven from this case, several important questions highlighting the complexity of decision-making in the local treatment of melanoma BMs can be discussed.

To date, there is no consensus on whether melanoma patients presenting with BM should undergo local treatment such as SRS additionally to systemic treatment (9), although brain control rates of dual immunotherapy or TKIs alone are only moderate. The brain control rate in the CheckMate 204 study, examining nivolumab plus ipilimumab in patients with melanoma BMs, was approximately 55% in asymptomatic patients and 20% in symptomatic patients (45). Regarding BRAF/MEKi, the COMBI-MB study reported a cerebral response rate of 58%, although responses were typically short-lived, with a progression-free survival of 5.6 months (46). Problematically, progression under BRAF/MEKi is generally resistant to immunotherapy with an intracranial response rate of 4.8% and median progression free survival of 1.3 months (47). Similarly, in the BREAK-MB study evaluating cerebral tumor control with dabrafenib, control rates ranged between 50 and 55%,consistent regardless of prior local treatment for BMs (48, 49).

Cerebral progression can significantly affect the patient’s quality of life and prognosis, as highlighted in this case report and in trials such as the Sampson trial (3). Therefore, strategies increasing cerebral control are warranted and the addition, timing and sequence of local BM treatment needs to be better defined. Studies combining SRS with immunotherapy have shown promise with local cerebral control rates well above those observed in systemic treatments alone. For example, the study of Minitti et al. reported a 12-month control rate of 85% with SRS plus nivolumab and 70% with SRS plus ipilimumab (50). A clinical neurological improvement after SRS occurred in 63% of patients with pre-existing neurological symptoms. Symptomatic radiation-induced brain necrosis occurred in 15% of patients. Similarly, data from Vaios et al. revealed 12-month SRS control rates of 95%, 92%, and 88% (respectively concomitant double immunotherapy, single immunotherapy, or no systemic treatment) (51). These findings underscore the potential benefits of combining SRS with immunotherapy with an acceptable toxicity profile and need to be further explored and integrated into modern decision-making.

Another argument in favor of early SRS treatment for BM could be applied to our patient, who eventually succumbed to disseminated leptomeningeal carcinomatosis, without extra-neurological disease progression. Surgery for a single brain metastasis can improve overall survival and quality of life (52) but also carries a risk of leptomeningeal dissemination, around 20% depending on the study (53). In this case, although speculative, leptomeningeal carcinomatosis may have been facilitated during brain surgery in 2020 and might have been avoided if SRS had been performed upon diagnosis of the first BM, instead of immunotherapy only, which did not lead to intracranial response.

**Figure 3:**
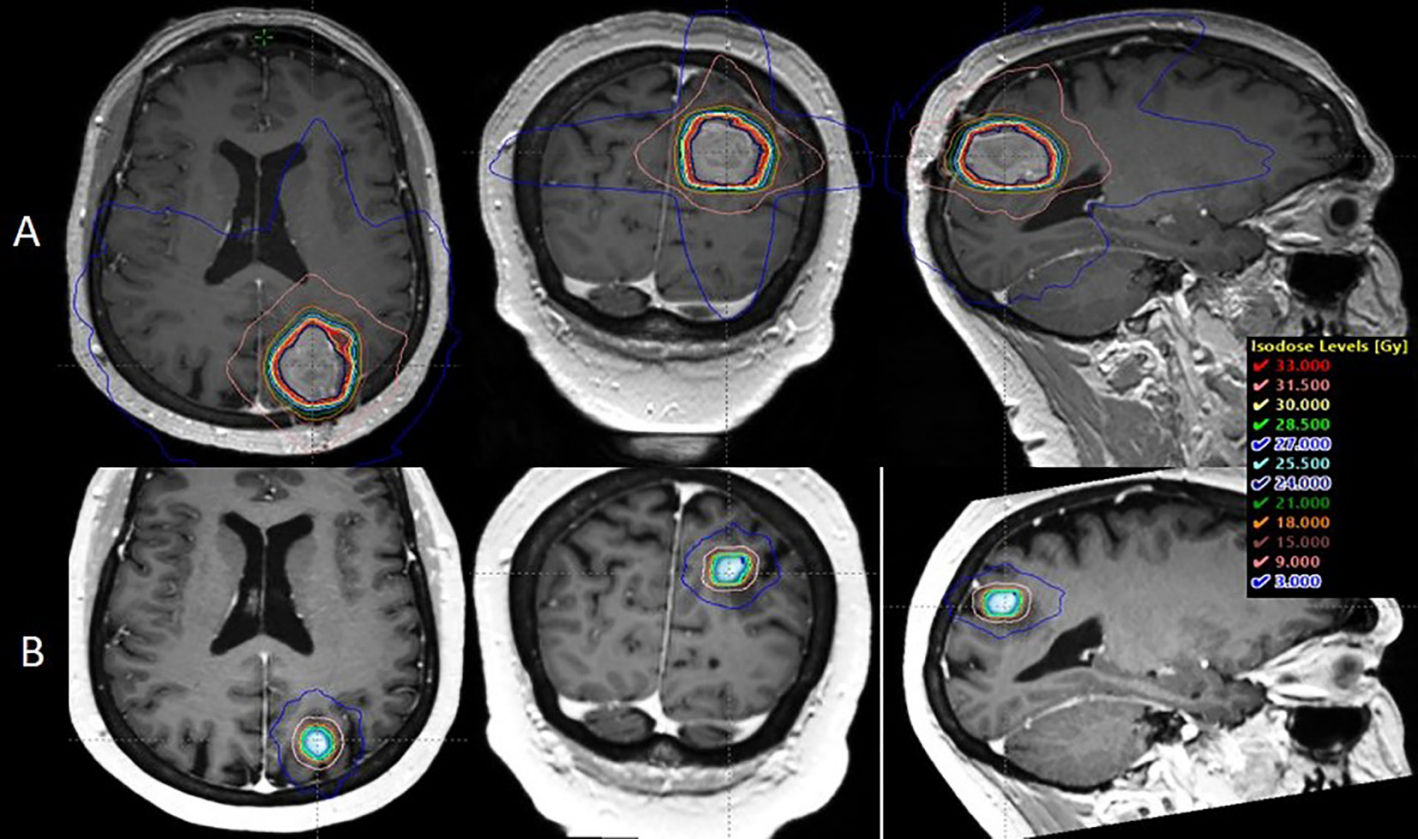
Dosimetric comparison between the post-operative radiotherapy performed in the case report and a simulation of single fraction 20 Gy SRS if delivered at first BM diagnosis: **(A)** post-operative context on the 17.5 cc cavity: Brain Dmean = 3. 001 Gy, V12 Gy = 49.13 cc. **(B)** exclusive radiotherapy context on the 1 cc BM, Brain Dmean = 0.434 Gy, V12 Gy = 2.98 cc.

We also performed a dosimetric simulation to illustrate the potential gain if SRS had been delivered at the time of the first diagnosis of the ultimately resected BM, which initially measured 1cm. Besides sparing the patient brain surgery and the increased risk of leptomeningeal dissemination, brain integral radiation dose would have been steeply reduced, as well as risk of RT-related adverse events (**Figure 3**). As an additional advantage for this individual case, at the time of the first BM diagnosis, the patient was receiving immunotherapy, suggesting that tolerance to systemic treatment combined with SRS might have been better than the one seen when she was under TKIs (54). However, it must be underlined that these scenarios are hypothetical and assume the good tolerance seen in the re-irradiation with concomitant TKIs was due to concomitant corticosteroid treatment.

Another lesson from our case report is that withholding efficient local treatment out of caution may be reasonable but comes with the risk of progression. Indeed, very early PP was the reason the patient did not receive a curative dose of RT on one out of the three lesions, which eventually progressed. Although this progression was not the cause of death, failure to locally control BM can lead to significant adverse effect in cancer patients.

It therefore becomes urgent to optimally integrate existing treatment options by taking into account both their efficacy and safety and better position SRS, a highly safe and effective modality within the time frame of the disease evolution of the individual patient. Within this frame, underdiagnosed rare adverse events, such as very early PP merit attention and should be reported systematically. Ongoing trials will provide valuable insight into the optimal treatment of melanoma BMs, such as the randomized, multicenter phase III USZ-STRIKE trial (NCT05522660), investigating the role of SRS in addition to standard systemic treatment for patients with metastatic melanoma or newly diagnosed metastatic NSCLC and asymptomatic or oligo-symptomatic BMs.

In conclusion, this case report highlights the first instance of ultra-early symptomatic PP after SRS in a patient under concurrent BRAF/MEKi therapy. This report and its discussion underscore the importance of particular vigilance when using TKIs concomitantly with SRS, as well as the uncertainties associated to the use of emerging treatments. Further research is warranted regarding the potential interactions between RT and novel systemic agents as well as their optimal administration sequence. Positioning local treatment modalities through emerging systemic treatments is an important priority of clinical research, which will ultimately allow improve outcomes, including quality of life. Despite this rare complication, this case prompts the discussion of the potential benefits of early SRS in the management of BMs, which merits investigation in academic clinical trials.

## Data Availability

The datasets presented in this article are not readily available because this is a case report and due to legal and ethical issues, patient level data used are unavailable (medical confidentiality). Requests to access the datasets should be directed to the corresponding author: edouard.romano@chuv.ch.
